# How to make better use of scientific knowledge for cancer prevention

**DOI:** 10.1002/1878-0261.12858

**Published:** 2020-12-02

**Authors:** Pekka Puska

**Affiliations:** ^1^ Finnish Institute for Health and Welfare (THL) Helsinki Finland

**Keywords:** cancer, evidence, implementation gap, prevention

## Abstract

Cancer prevention research has produced profound scientific knowledge that has led to the development of several evidence‐based prevention strategies. But do these research outcomes lead to preventive action in real life? Many factors contribute to the so‐called ‘implementation gap’ between prevention recommendations and their application and adherence, including individual actions and behaviour, health service structures and political actions. This article discusses factors underlying the implementation gap in both clinical‐ and population‐based prevention. Understanding how these factors contribute to the implementation gap is important for planning successful cancer prevention strategies, as well as generally achieving disease prevention.

## Graphical abstract

Scientific research has provided a solid basis for cancer prevention, concerning both high‐risk individuals and populations. However, several factors hinder implementation of existing evidence. This article discusses current implementation gaps and the ways to overcome them to apply available knowledge for cancer prevention.

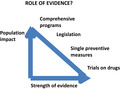

**Abbreviations**
GBD: Global Burden of Disease
NCDs: noncommunicable diseases

## 1 Introduction

Cancer is among the leading burdens of disease and causes of death worldwide. According to the 2017 Global Burden of Disease (GBD) study, 17% of all deaths worldwide were due to neoplasms [1]. Cancer was listed as the second cause of death after cardiovascular disease (24%). Thus, cancer and other noncommunicable diseases (NCDs) are in key positions among priorities for global public health. Furthermore, from 1990 to 2017 there has been a 38% increase in cancer mortality [1]. In Europe, cancer is responsible for ~ 25% of all deaths, and this percentage is expected to increase to 32% by 2040 [2].

During the last few decades, there has been extensive scientific research on the causes and aetiology of human cancers. Cancer research has involved work on the genetic and molecular mechanisms of cancers, clinical treatment, the epidemiology of cancers and their risk factors, as well as on the behavioural, social and economic aspects of cancer prevention and treatment. Notably, the largest body of cancer research focuses on aspects other than prevention.

Prevention of cancers concerns both prevention of specific types of cancer and general prevention of cancer in the population. The European Code against Cancer provides a general framework for cancer prevention in the European population [3]. As many cancers share common risk factors with other NCDs, cancer prevention in fact deals much with the general prevention of NCD. As global burden of NCD has been increasing during the last few decades, WHO has been actively engaged with issues of NCD prevention and control. The WHO Global Strategy on NCD Prevention and Control that was issued in 2000 [4] has been a landmark for global work in this area.

The global work for NCD prevention and control has thereafter been much strengthened by further decisions of the World Health Assembly and was also supported by UN meetings that have resulted in respective political declarations. Specifically, following the 2011 high‐level UN meeting, WHO adopted its first Global Action Plan for the Prevention and Control of NCDs, with concrete objectives and targets aiming at a reduction by 25% in the risk of premature mortality from NCDs (cardiovascular disease, cancer, diabetes and chronic respiratory disease) by 2025 [5]. This work was further strengthened by subsequent UN meetings and declarations. In 2015, the UN declaration linked NCD prevention and control with the Global Sustainable Development Goals and adopted a target of reducing premature NCD mortality by 30% by 2030 [6].

## 2 Evidence for action

**Fig. 1:**
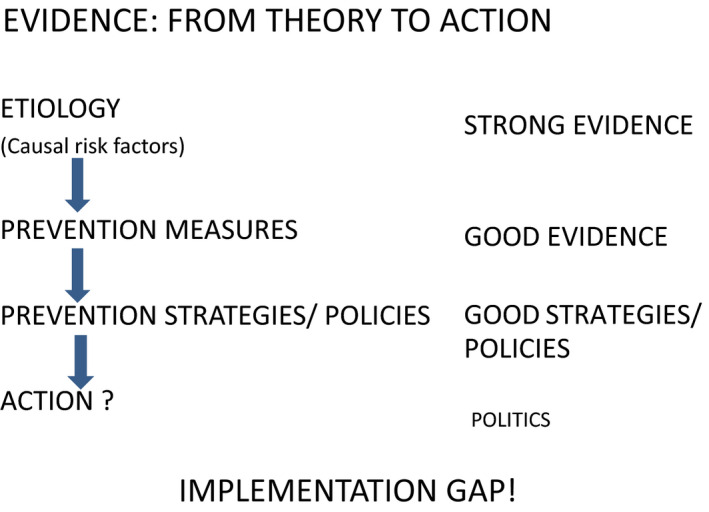
Strength of the evidence: from theory to action.

Knowledge accumulated through current cancer research provides very strong evidence for developing effective cancer prevention strategies. It has often been said that the problem is not ‘what to do’, but ‘how to do it’. Before we discuss whether research‐based evidence really leads to action, we must define types of evidence. There are several types of evidence concerning cancer prevention (Fig. 1).

The first type is derived from the combination of molecular research, observational studies and clinical trials on the causal role of many risk factors of cancer and is extremely strong. The second type of evidence, relating to the effects of single preventive interventions, is also quite potent. The third type of evidence, which is derived from comprehensive health promotion programmes and policies, is, however, less strong when compared with the other two types, as its outcomes may vary depending on the cultural environment. Several ‘evidence‐based’ strategies based on collective evidence from basic, clinical and prevention research as well as on existing policies have been developed. However, political implementation of this scientific knowledge remains challenging.

**Fig. 2:**
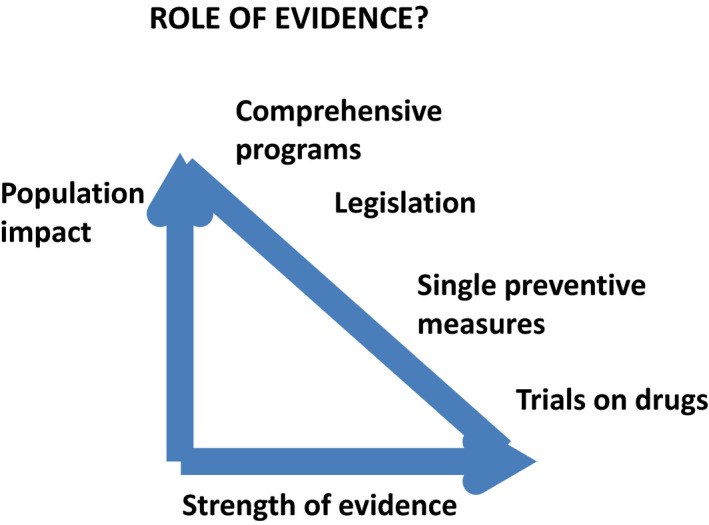
Population impact of various interventions and the strength of evidence.

There is another important consideration concerning the implementation of evidence into action (Fig. 2). While the evidence for lifestyle interventions in the population may be soft, evidence for clinical interventions, such as drug‐based interventions, can be very strong. However, the impact of drug‐based interventions on the population level can be very narrow compared with the potential of population‐based lifestyle interventions. Furthermore, the cost of extensive drug‐based or other clinical interventions may be high compared with the low cost of policy interventions for prevention of cancers and other NCDs in the population.

## 3 Types of prevention

Based on some basic principles we can distinguish between two types of prevention: high‐risk, or clinical‐based, prevention; and population‐based, or public health, prevention.

High‐risk prevention targets individuals with established high risk and aims at reducing risk by the use of drugs or other clinical measures, as well as through behavioural counselling. This strategy may be very important at the individual level. It involves costs, but the higher the risks of disease, the more beneficial the cost‐effectiveness ratio. However, as high‐risk prevention usually targets a relatively small group of people, the impact on population level can be low. The larger the group is, the higher are the costs involved.

The population approach does not involve detection of high‐risk individuals in the population. Through broad health policies, the aim is to promote healthy lifestyle changes in the entire population. A classic example is the implementation of legislation and policies on tobacco use that have resulted in major reductions in smoking and in reductions in rates of cancer and other NCDs in the population, with very limited costs.

**Fig. 3:**
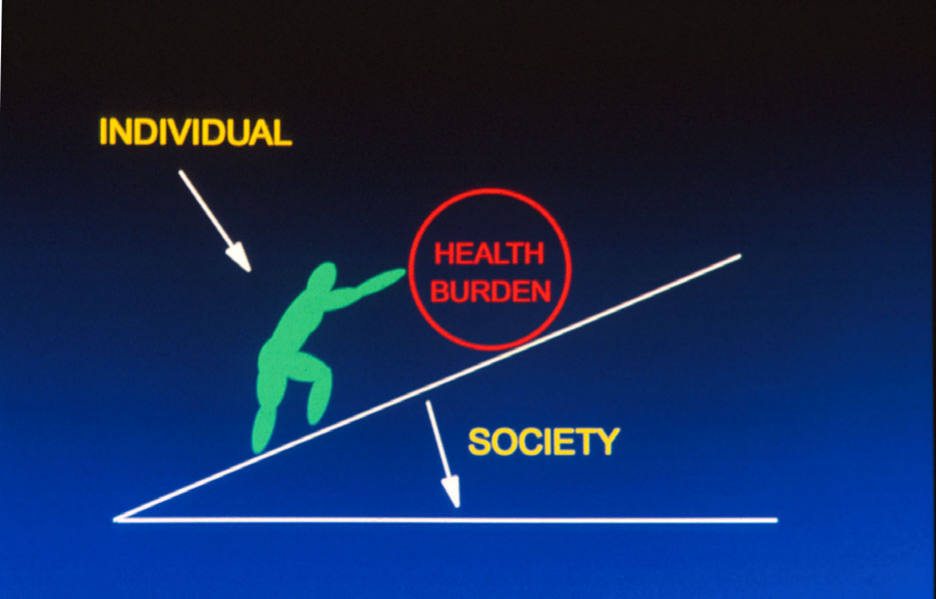
Difference between individual high‐risk prevention and population‐based societal prevention.

The difference and nature of the two types of prevention are illustrated in the Fig. 3. The high‐risk approach tries to help or empower the individual to cope with the health burden, that is, to reduce the risk, often through challenging behavioural changes. The population approach tries to make societal changes that would lower the slope, so that healthier behaviours for people are easier in society. This reflects the common phrase ‘Make the healthy change, the easy change’, developed after the Ottawa Health Promotion Charter [7].

Obviously, both prevention types have useful applications and they are by no means mutually exclusive. High‐risk prevention helps individuals and is the primary focus of health services, whereas population‐based prevention helps populations and depends on political decisions and other societal measures. Population‐based interventions also reach patients, and patients are often opinion leaders in disease‐related issues in the community.

## 4 How to enhance the use of evidence in high‐risk prevention

High‐risk individuals are usually identified during health service visits (‘opportunistic screening’) or through special population screenings. There are many good reviews on principles for screening in general or for cancers specifically [8, 9].

After identification of the cancer risk, it remains optimally to implement existing knowledge for the preventive action. Several national and international consensus statements and guidelines are available to guide the work of health services and health professionals. IARC continuously publishes handbooks of cancer prevention as background for the current state of knowledge [at https://handbooks.iarc.fr/ (last accessed 10 July 2020)].

The extent of adherence to the latest guidelines depends on the structure of health services and the training and supervision of health practitioners. In addition to medical education, continuous education and training incorporating new knowledge is important. A good information system, preferably an electronic one, is needed for standardizing risk documentation and assessment, as well as for monitoring of progress – and, ideally, for identifying needed actions.

Much of the actual benefit of high‐risk prevention strategies, however, depends on the individuals themselves. This mainly concerns compliance with or adherence to health guidelines and medical advice. A lot of research has been carried out on this topic and many good summaries and guidebooks have been published; for instance, in 2003 WHO published an important report on adherence [10]. This provides a good description of the various aspects of compliance by the individual, whether for drug‐based or for behavioural interventions.

The WHO report describes how adherence is influenced by several factors, including social and economic factors, the functioning of preventive services, type of disease and risk in question, the advised measure and the subject herself/himself. The report illustrates how compliance is an important modifier of effectiveness and of the impact of existing medical knowledge. The report also recommends systematic use of scientific knowledge, but also points out that patient‐tailored interventions are needed. Behavioural research provides lessons for patient counselling. It is important to realize that it is often not sufficient merely to give information and recommendations to the individual. Individuals need different kinds of assistance and support. Blaming them for poor adherence is not fruitful. Instead, the problematic of the healthcare system and counselling should be examined.

## 5 How to enhance the use of evidence in population‐based prevention?

As mentioned earlier, the increase in available evidence on the causal role of many risk factors in cancer development, or in other NCD development, has led to a growing number of evidence‐based preventive strategies, both locally and internationally. In this case, the key question is often the implementation of these strategies, not the existing evidence *per se*. Many strategies remain reports stored on shelves and very rarely implemented. This problem is often called ‘the implementation gap’.

The underlying causes of the implementation gap are numerous: e.g. inertia in change, cultural aspects, environmental factors, economic issues and interests, lobbying and professional interests. Preventive actions often require political decisions, sometimes concerning areas outside the health sector. This is emphasized in the ‘Health in All Policies’ approach [11, 12]. However, decision makers in sectors other than the health sector are often not motivated by health‐focused arguments, especially if they conflict with the needs of their sector.

Of course, strong evidence and good strategies are always key, but often these are not sufficient for the needed political decisions on prevention. This is because the health evidence, even if compelling, is not the only consideration for politicians. Other aspects often concern funding, but also issues of feasibility, economics, employment, individual, commercial rights, etc. Often other interest groups oppose health data‐driven policies in the traditional or social media with conflicting messages serving their needs.

Although strong scientific evidence is usually seriously considered, the major driving forces for political decisions are opinions, intentions and behaviours of the population. For the politicians in democracy ‘the voter is king’ – for the private sector ‘the consumer is king’.

Thus, to promote effective political decisions for prevention, especially for more difficult issues, it is important to mobilize the population. To this purpose, promotion of a good health paradigm through the building of civil society coalitions and the innovative use of media, among others, is important. The messages should, if possible, be linked not only with health‐related benefits but also with other positive outcomes of prevention, including broader NCD impact, economic savings, improved quality of life, environmental benefits and sustainable development.

The existence of a good institutional base is also important for promoting decision making on prevention. Universities perform valuable research work. However, public health institutes, both national and international, such as IARC, are of key importance, as they have close links with decision makers, provide practical experience and have the capacity to monitor progress [13]. Monitoring of national trends in risk factors and risk‐related behaviours is a strong tool for national prevention, as ‘what gets measured, gets talked about’.

Direct contact and communication with politicians is often not easy; they have extremely busy schedules and numerous stakeholders try to influence them, often with conflicting messages. The best approach is personal meetings, arranged by their peers, if possible. Communicated messages should be short, with clear and concise recommendations and justification, and counter arguments must be anticipated. Messages through the traditional or social media have maximum impact on politicians when they come from their own constituency or their support groups.

## 6 More prevention research is needed

There is a general consensus that prevention of cancer is both possible and desirable, at least in some cases, but the potential of prevention strategies is much underused. Wild *et al*. [2] note that although a high proportion of cancers in Europe are attributable to modifiable factors, the majority of cancer research focuses on basic science and clinical research. Even within the cancer prevention field, most research focuses on identification of causal risk factors rather than implementation research.

Further research is needed on the methods, process and practices of prevention in various environments, so that the implementation gap is bridged and prevention strategies become successful. We need to learn more about how research evidence can be translated into both clinical practice and population‐based prevention. Translational research in medicine often refers to improving the process from laboratory to bedside, but also to translating research results for the benefit of the population, that is, improving population‐based prevention. As said, much attention is given to research to identify new risk factors, and less attention and resources to the question of how better to apply the solid and well‐known evidence on the strong causal risk factors.

Translational research and prevention research involve many disciplines in addition to medicine and epidemiology. Behavioural and economic research is needed in patient‐based prevention to support better compliance and to learn about the cost‐effectiveness of different prevention options. Cost‐effectiveness is also an important aspect in population‐based prevention, and this also calls for social and political research.

Prevention research focusing on strategies to overcome the many causes of the implementation gap is thus valuable. At the same time, it should be realized that, especially in the field of population‐based prevention, the much‐needed political action is more challenging than simply the application of research results. In the political world, even the most convincing research results only make up the background, rather it is the initiatives to influence political decisions that constitute the ‘state of political art’.

## 7 Conclusions

The long‐term value of research, research‐driven data and the related evidence for cancer prevention – or public health in general – is undisputable. However, it is also important to realize that successful implementation of evidence is a complicated process. Understanding current shortcomings of both high risk‐based prevention and population‐based preventive policies is important for future success. This is also an important topic for further research.

It is often said that on a policy level the most effective preventive measures are the hardest, while the least effective are the easiest. The public health community should remember the old saying that ‘no pain – no gain’, or ‘no struggle – no progress’!

## Conflict of interest

The authors declare no conflict of interest.

## Author contribution

Sole product of the author.
